# Diverse Plant-Associated Pleosporalean Fungi from Saline Areas: Ecological Tolerance and Nitrogen-Status Dependent Effects on Plant Growth

**DOI:** 10.3389/fmicb.2017.00158

**Published:** 2017-02-06

**Authors:** Yuan Qin, Xueyu Pan, Christian Kubicek, Irina Druzhinina, Komal Chenthamara, Jessy Labbé, Zhilin Yuan

**Affiliations:** ^1^Institute of Subtropical Forestry, Chinese Academy of ForestryHangzhou, China; ^2^Research Area Biochemical Technology, Institute of Chemical Engineering, TU WienVienna, Austria; ^3^Biosciences Division, Oak Ridge National Laboratory, Oak RidgeTN, USA

**Keywords:** Pleosporales, dark septate endophytes, halophytes, organic nitrogen, symbiosis

## Abstract

Similar to mycorrhizal mutualists, the rhizospheric and endophytic fungi are also considered to act as active regulators of host fitness (e.g., nutrition and stress tolerance). Despite considerable work in selected model systems, it is generally poorly understood how plant-associated fungi are structured in habitats with extreme conditions and to what extent they contribute to improved plant performance. Here, we investigate the community composition of root and seed-associated fungi from six halophytes growing in saline areas of China, and found that the pleosporalean taxa (Ascomycota) were most frequently isolated across samples. A total of twenty-seven representative isolates were selected for construction of the phylogeny based on the multi-locus data (partial 18S rDNA, 28S rDNA, and transcription elongation factor 1-α), which classified them into seven families, one clade potentially representing a novel lineage. Fungal isolates were subjected to growth response assays by imposing temperature, pH, ionic and osmotic conditions. The fungi had a wide pH tolerance, while most isolates showed a variable degree of sensitivity to increasing concentration of either salt or sorbitol. Subsequent plant–fungal co-culture assays indicated that most isolates had only neutral or even adverse effects on plant growth in the presence of inorganic nitrogen. Interestingly, when provided with organic nitrogen sources the majority of the isolates enhanced plant growth especially aboveground biomass. Most of the fungi preferred organic nitrogen over its inorganic counterpart, suggesting that these fungi can readily mineralize organic nitrogen into inorganic nitrogen. Microscopy revealed that several isolates can successfully colonize roots and form melanized hyphae and/or microsclerotia-like structures within cortical cells suggesting a phylogenetic assignment as dark septate endophytes. This work provides a better understanding of the symbiotic relationship between plants and pleosporalean fungi, and initial evidence for the use of this fungal group in benefiting plant production.

## Introduction

As intimate partners of plants, many groups of fungi can establish associations with roots and seeds and thereby facilitate plant growth and increase stress tolerance (Ernst et al., 2003; Rodriguez et al., 2009; de Zelicourt et al., 2013). Plant-associated mycobiota comprise taxonomically diverse members, mainly including arbuscular mycorrhizal fungi (AMF), ectomycorrhizal fungi (EMF) and a number of ascomyceteous and non-mycorrhizal basidiomycetous fungi (NMF) (Gardes and Dahlberg, 1996; Khidir et al., 2010; Zuccaro et al., 2014). Mycorrhizal symbioses have been extensively described due to their important role in improving plant nutrition and stress tolerance (Evelin et al., 2009). Despite accumulating evidence that plant roots can host many more non-mycorrhizal endophytes than previous thought (Vandenkoornhuyse et al., 2002; Porras-Alfaro et al., 2008; Toju et al., 2013), the ecological significance of NMF plant associations are poorly understood.

Plant fungal endophytes have been categorized into four groups on the basis of a series of criteria including host colonization pattern, transmission model (vertical transmission *via* host seeds and horizontal transmission *via* soil- or air-borne spores) and fitness benefits (Rodriguez et al., 2009). Notably, class 2 fungal endophytes can establish habitat-adapted symbiosis and confer specific stress tolerance to the host plant in different extreme habitats (Rodriguez et al., 2008; Redman et al., 2011). Similarly, dark septate endophytes (DSEs) are considered to be class 4 endophytes and form melanized hyphae and microsclerotia-like structures in roots (Knapp et al., 2015; Yuan et al., 2016). DSEs are the dominant root-associated fungi and more frequent than AMFs from plants grown in extreme environments (e.g., salinity and drought) (Porras-Alfaro et al., 2008; Newsham et al., 2009). Some root opportunistic and rhizospheric fungi can also induce systemic resistance against crop diseases (Shoresh et al., 2010; Druzhinina et al., 2011; Jogaiah et al., 2013) and improve abiotic stress tolerance (McLellan et al., 2007). These findings underscore the importance of NMF in mediating plant productivity. However, it is generally poorly understood how plant-associated fungi are structured in in extreme conditions, and if so, to what extent they contribute to improving plant performance.

The Pleosporales order is considered to be among the largest class within the class Dothideomycetes (Ascomycota) (Phookamsak et al., 2014; Tibpromma et al., 2015). Some genera of this order comprise ecologically important plant endophytes, including numerous DSEs (Hamayun et al., 2009; Knapp et al., 2015). Knapp et al. (2012) demonstrated that pleosporalean fungi occurred in all plant species in semi-arid grasslands of North America. Furthermore, microscopic analysis of the grass *Bouteloua gracilis* revealed that the fungal community of roots was dominated by a novel DSE belonging to Pleosporales (Porras-Alfaro et al., 2008). Large-scale culture-based surveys show that some fungal genera of the Pleosporales are common endophytes in both coastal and inland arid soils (Kageyama et al., 2008; Maciá-Vicente et al., 2008). Consequently, we surmise that pleosprolean fungi are generalist endophytes common within adverse environments. However, their basic physiological characters and potential ecological significance has received only very limited attention.

In this work, a wide range of pleosporalean fungi were isolated from the rhizosphere, roots and seeds of halophytic plants in China. We then determined their phylogeny, sensitivity to diverse environmental stresses, and their ability to utilize various substrates of nitrogen. Further, we investigated their effects on plant growth in the presence of organic and inorganic nitrogen.

## Materials and Methods

### Study Site and Sampling

**FIGURE 1:**
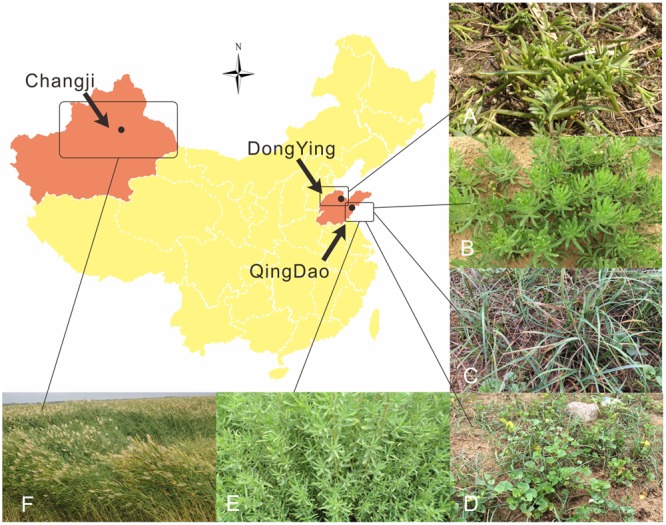
**Map of collection sites and photos of plant samples in this study**.

A total of six halophytes were collected at three sampling sites in China. In August 2011, the healthy roots of *Phragmites australis* (family Poaceae) were collected at the inland saline and arid soil of Changji, XinJiang Province (N 44°29′, E 87°93′), northwest of China. In July of 2014, roots and rhizosphere soils of *Suaeda salsa* were collected from the coastal region at the mouth of the Yellow River in DongYing, ShanDong Province (N 37°23′, E 118°55′). Furthermore, the rhizosphere soil, intact roots of *S. salsa* (family Amaranthaceae), *P. australis, Calystegia soldanella* (family Convolvulaceae), *Carex scabrifolia* (family Cyperaceae), *Kochia scoparia* (family Amaranthaceae) and *Messerschmidia sibirica* (family Boraginaceae) were collected from a saline coastal sandy soil in QingDao, ShanDong Province (N 35°51′, E 120°02′) in 2015. The plant species selected are considered to be good bio-indicators of saline environments. A map of collection sites and photos of plant samples was provided in **Figure 1**.

### Isolation of Endophytic and Rhizospheric Fungi from Six Halophytes

Fungi associated with rhizosphere soils (within a 1-mm vicinity of the roots) were isolated using the traditional serial dilution technique (Jogaiah et al., 2013). For collecting the soil samples, roots with adhering soil were mixed with 40 ml sterile phosphate buffer solution (100 mM PBS, pH = 7.2) in 50-ml tubes (BD Falcon). Tubes were vortexed at high speed for 5 min using a Vortex Genie 2 (Mo Bio Laboratories Inc., USA), allowing the release of most of the rhizosphere soils. The root samples were removed and soil suspensions were centrifuged for 15 min at 10°C at 6000 *g* to pellet the rhizosphere soils. Then, 1 g of each soil sample was re-suspended in 99 ml PBS and 10-fold serial dilution was made from the suspension (10^-3^ to 10^-5^). Dilution of 10^-4^ and 10^-5^ were used to isolate fungi. 100 μl of each diluted suspension was spread evenly onto Czapek’s agar plates supplemented with 0.05 g/L streptomycin sulfate and 0.02 g/L tetracycline hydrochloride to eliminate bacterial contamination. The composition of the medium was as follows: sucrose 30 g, NaNO_3_ 2.0 g, K_2_HPO_4_ 1.0 g, MgSO_4_^.^7H_2_O 0.5g, KCl 0.5 g, FeSO_4_^.^H_2_O 0.01g, agar 20 g and 1 liter of distilled water, pH 5.6. All plates were incubated at 25°C in darkness for at least 1 week until colonies appeared. Colonies were picked up and transferred to fresh potato dextrose agar (PDA) plates for purification.

For isolating fungal endophytes, roots and seeds (excised from the utricles) were heavily surface sterilized by immersion in ethanol (75%, v/v) for 30 s, and then soaked in 2.0% NaClO (v/v) for 5–10 min depending on the type of tissues, followed by 90% ethanol (v/v) for 30 s to remove the residual NaClO. Finally, sterilized distilled water was used to rinse the samples at least five times. The roots were dried with sterile filter paper, cut into 3–5 mm segments (for seeds cut into two halves), and placed on 2% malt extract agar (MEA, Oxoid) plates supplemented with antibiotics as mentioned above. Totally, 70 seed segments from *S. salsa* and approximately 160 root segments from each plant species were used for isolation. The plates were incubated for 2 weeks at 20°C in the dark, after which emerging hyphae developed from the tissue fragments were transferred to PDA for purification.

### DNA Extraction, PCR Amplification and Sequencing

The mycelia were scraped from the PDA plates and transferred to a sterile 1.5 ml microcentrifuge tube. Fungal DNA was extracted using DNeasy Plant Mini Kit (Qiagen) according to the manufacturer’s protocol. Four primer pairs, ITS1F and ITS4 (White et al., 1990), NS1 and NS4 (White et al., 1990), LR0R and LR5 (Vilgalys and Hester, 1990) as well as EF1-983F and EF1-2218R (Rehner and Buckley, 2005) were used for the amplification of internal transcribed spacer (ITS) of the rDNA cluster, partial 18S rRNA (small subunit, SSU) and 28S rRNA (large subunit, LSU), and partial transcription elongation factor 1-α (*tef1)* gene, respectively. The final volume of the PCR reaction was 50 μl which contained 2 μl template DNA (20–50 ng), 0.5 μl of each forward and reverse primer (50 μM), 25 μl 2x Taq MasterMix (Cwbio, Beijing), and 22 μl sterile deionized water.

The PCR reaction consisted of the following steps: a pre-denaturation at 94°C for 4 min, followed by 35 cycles of denaturation at 94°C for 40 s, annealing at 55°C for 50 s and elongation at 72°C for 1 min (2 min for LSU and SSU), and final extension at 72°C for 10 min. For *tef1* amplification, we used a touchdown PCR cycle that started with an annealing temperature of 66°C in the first cycle, reducing by 1°C in each successive cycle over the next nine cycles until it reached 56°C, which was used in the remaining 30 cycles. All purified PCR products were sent to Shanghai Sangon Biological Engineering Technology and Services Co., Ltd (Shanghai, China) and sequenced with the above primer pairs. All sequences from this study have been deposited in the GenBank database under the accession numbers presented in **Table 1**. ITS sequences were subjected to BLASTn searches^1^ for initial identification.

**Table 1:** The list of pleosporalean isolates obtained in this study and their corresponding GenBank accession numbers of different loci (ITS, LSU, SSU, and *tef1*).

Isolate no.	Identity	Host	Isolation source	Collected site	GenBank accession no.			
					ITS	LSU	SSU	*tef1*
CYTC-R-5	*Phaeosphaeriaceae* sp.	*Carex scabrifolia*	Root	QingDao	KU991882	KU991906	KX894737	KX894764
DF-R-1	*Phaeosphaeriaceae* sp.	*Kochia scoparia*	Root	QingDao	KU991883	KU991907	KX894738	KX894765
DF-R-3	*Phaeosphaeriaceae* sp.	*Kochia scoparia*	Root	QingDao	KU991884	KU991908	KX894739	KX894766
DF-R-7	*Phaeosphaeriaceae* sp.	*Kochia scoparia*	Root	QingDao	KU991885	KU991909	KX894740	KX894767
DF-R-9	*Didymosphaeriaceae* sp.	*Kochia scoparia*	Root	QingDao	KU991886	KU991910	KX894741	KX894768
DW-R-1	*Phaeosphaeriaceae* sp.	*Calystegia soldanella*	Root	QingDao	KU991887	KU991911	KX894742	KX894769
DW-R-3	*Saccharicola* sp.	*Calystegia soldanella*	Root	QingDao	KU991888	KU991912	KX894743	KX894770
DW-R-4	*Paraconiothyrium* sp.	*Calystegia soldanella*	Root	QingDao	KU991889	KU991913	KX894744	KX894771
JP-R-2	*Alternaria* sp.	*Suaeda salsa*	Root	QingDao	KU991890	KU991914	KX894745	KX894772
JP-R-4	*Phaeosphaeriaceae* sp.	*Suaeda salsa*	Root	QingDao	KU991891	KU991915	KX894746	KX894773
JP-R-44	*Didymosphaeriaceae* sp.	*Suaeda salsa*	Root	DongYing	KJ125522	KJ125523	KX894748	KX894775
JP-R-6	*Didymosphaeriaceae* sp.	*Suaeda salsa*	Root	QingDao	KU991892	KU991916	KX894747	KX894774
LW-7	*Lentitheciaceae* sp.	*Phragmites australis*	Root	XinJiang	KU991893	KU991917	KX894749	KX894776
LW-R-2	*Epicoccum* sp.	*Phragmites australis*	Root	QingDao	KU991894	KU991918	KX894750	KX894777
LW-R-3	*Paraconiothyrium* sp.	*Phragmites australis*	Root	QingDao	KU991895	KU991919	KX894751	KX894778
R(2015)-25	*Curvularia* sp.	*Suaeda salsa*	Root	DongYing	KU991898	KU991922	KX894754	KX894780
R(2015)-29	*Alternaria* sp.	*Suaeda salsa*	Root	DongYing	KU991899	KU991923	KX894755	KX894781
R(2015)-4	*Alternaria* sp.	*Suaeda salsa*	Root	DongYing	KU991897	KU991921	KX894753	KX894782
R20	*Phaeosphaeriopsis* sp.	*Suaeda salsa*	Root	DongYing	KU991896	KU991920	KX894752	KX894779
RS1-8	*Alternaria* sp.	*Suaeda salsa*	Rhizosphere	DongYing	KU991900	KU991924	KX894756	KX894783
RS-A-34	*Phaeosphaeriaceae* sp.	*Suaeda salsa*	Rhizosphere	DongYing	KU991901	KU991925	KX894757	KX894784
RS-A-88	*Phaeosphaeriaceae* sp.	*Suaeda salsa*	Rhizosphere	DongYing	KU991902	KU991926	KX894758	KX894785
RS-C-28	*Phaeosphaeriaceae* sp.	*Suaeda salsa*	Rhizosphere	DongYing	KU991903	KU991927	KX894759	KX894786
RS-JP-2	*Phaeosphaeriaceae* sp.	*Suaeda salsa*	Rhizosphere	QingDao	KU991904	KU991928	KX894760	KX894787
seed1	*Preussia* sp.	*Suaeda salsa*	Seed	DongYing	KU869522	KU991929	KX894761	KX894788
seed6	*Phaeosphaeriaceae* sp.	*Suaeda salsa*	Seed	DongYing	KU869527	KU991930	KX894762	KX894789
SYC-R-4	*Didymosphaeriaceae* sp.	*Messerschmidia sibirica*	Root	QingDao	KU991905	KU991931	KX894763	KX894790

### Molecular Phylogeny of the Plant-Associated Pleosporalean Fungi

Preliminary ITS sequence-based identification supports the existence of a wide range of pleosporalean taxa. Thus, we aim to infer their phylogeny using three loci (SSU, LSU, and *tef1*), which have been used to give sufficient phylogenetic resolution within the Pleosporales (Tanaka et al., 2015), to provide insights into the relationships between our isolates and known pleosporalean fungi. To construct the phylogenetic tree, we retrieved the corresponding gene sequences from recently published papers focusing on the molecular phylogeny of the Pleosporales (Lumbsch and Lindemuth, 2001; de Gruyter et al., 2006, 2010; Ariyawansa et al., 2014; Phookamsak et al., 2014; Knapp et al., 2015; Phukhamsakda et al., 2015). A concatenated set of three genes from the strains listed in **Table 1** and Supplementary Table S1, was used to create an alignment using muscle v3.8.425 (Edgar, 2004) tool integrated in AliView (Larsson, 2014). The conserved concatenated alignment based on nucleotides containing 2604 characters was subjected to Bayesian analysis using program MrBayes v3.2.5 (Huelsenbeck and Ronquist, 2001). The chain was run for 5 million generations by applying Generalized time reversible substitution model (Waddell and Steel, 1997). Two simultaneous, completely independent analyses starting from different random trees were run, using three heated chains and one “cold” chain. Once the analysis was finished, 37500 trees were summarized after discarding the first 25% of obtained 50,000 trees, and a consensus tree was obtained. Branch color corresponding to the posterior probability percentage of each clade was generated using FigTree v1.4.2^2^. Further image improvement was performed in CorelDraw Graphics Suite X8.

### Fungal Growth Responses to Environmental Stresses

To explore the adaptability of the pleosporalean fungi to adverse environmental conditions, their growth rates under various conditions (temperatures, pH, salt and osmolyte concentrations) were examined. Colony diameter was used as parameter. Mycelial plugs (5.0 mm in diameter) cut from 7-day-old PDA colonies were transferred to fresh PDA plates.

To evaluate fungal growth responses to different levels of temperatures and pH, the inoculated PDA plates were incubated at 10, 15, 20, 25, 28, and 30°C in the dark for 7 days. Similarly, fungi were cultured on PDA with a wide range of pH gradients (ranging from 6 to 11). The pH values were measured with a Mettler Toledo pH meter. All plates were incubated at 25°C. The salt stress was induced by adding different concentrations of ionic osmolytes (NaCl and KCl), and the non-ionic osmotic stress was imposed by using sorbitol. First, the PDA medium was supplemented with salt (KCl and NaCl) at concentrations of 2, 4, 6, 8, 10, and 12% (w/v), respectively. In parallel, PDA medium with non-ionic osmotic treatment was prepared using sorbitol (0.2–2.0 M) (Nikolaou et al., 2009). All plates were incubated at 25°C. Three to five replicates were performed for each treatment. This experiment was terminated after 10 days. Colony diameters of these fungi were recorded. Values were means of replications and shown in Radar charts.

### Nitrogen Utilization Pattern of Pure Fungal Cultures

We used the modified Melin and Norkrans (MMN) free of nitrogen as the basal medium, to determine the ability of the fungi to utilize different inorganic and organic forms of nitrogen (Midgley et al., 2004; Bizabani and Dames, 2016). The nitrogen-free MMN medium contained (L^-1^): 5.0 g glucose, 0.30 g KH_2_PO_4_, 0.14 g MgSO_4_^⋅^H_2_O, 50 mg CaCl_2_, 25 mg NaCl, 3 mg ZnSO_4_, 12.5 mg ferric EDTA and 0.13 mg thiamine-HCl, pH 4.5 prior to autoclaving. For all treatments, a final N concentration was adjusted to 50 mg^⋅^L^-1^. The inorganic N source, ammonium [0.25 g^⋅^L^-1^ (NH_4_)_2_HPO_4_] and nitrate (0.36 g^⋅^L^-1^ KNO_3_), were separately added into the basal medium. A mixture of acidic, neutral, and aromatic amino acid amino acids [glutamine (Glu), glycine (Gly), valine (Val), leucine (Leu), and phenylalanine (Phe)], as well as Bovine Serum Albumin (BSA, N content 16%), were chosen as the organic N source. The two solutions were filter-sterilized with 0.45 and 0.22 μm Millipore filters and added into the autoclaved nitrogen-free basal medium separately. Three mycelial plugs of every fungus (5.0 mm in diameter) were cut with a sterile cork-borer from the edge of actively growing colonies on PDA plates, and then inoculated into 50 ml of the liquid medium in 250 ml flasks. Three replicates were prepared for each treatment. All the cultures were incubated in the dark, at 26°C and shaking at180 r/min. After 7 days, mycelia were collected by filtration through filter paper, and the growth of fungi was measured as dried mycelial biomass (mg).

### Plant-Fungal Co-culture Assay under Organic and Inorganic Nitrogen Conditions

We established the plant–fungal co-culture system to investigate the effects of fungal inoculation on plant growth. We also ask if the nitrogen status in the plant growth substrate will influence the outcome of plant–fungal interactions. In this case, both organic nitrogen in the form of amino acids and inorganic nitrogen in the form of ammonium nitrate (NH_4_NO_3_) and potassium nitrate (KNO_3_).

The gnotobiotic rice seedlings were generated using the following steps. The rice seeds (cultivar: zhongjiazao 17) were surface sterilized as mentioned above. Seeds were sown in Petri dishes (150 mm diameter) containing 1/5 Murashige and Skoog (MS) medium and incubated at 28 ± 1°C for 96 h. Thereafter, the germinated seeds without microbial contamination were transplanted into the sterile plastic container with 1/2 MS and placed on growth chamber with a photoperiod of 12 h of light/12 h darkness and temperature of 28 ± 1°C for 5 days (Rodriguez et al., 2008). Uniform sized seedlings were selected for inoculation.

To support the growth of these diverse pleosporalean fungi as well as maintain a moderate fungal growth rate, 2 g oatmeal. L^-1^ (Difco) as the carbon source was added into the 1/5 Murashige and Skoog (MS medium) (Mahmoud and Narisawa, 2013). Inorganic N sources (KNO_3_ and NH_4_NO_3_) were added to the basal medium before autoclaving (Finlay et al., 1992), while a mixture of five amino acids (Glu, Gly, Val, Leu, and Phe) was filter-sterilized with 0.22 μm Millipore filters and added to the autoclaved basal medium with a final concentration of 30 mg N.L^-1^ (Usuki and Narisawa, 2007). A total of 70 ml medium was poured into a sterile glass tube (25 mm diameter, 250 mm height). One fresh mycelial plug (5 mm in diameter) cut from colony margins was placed mycelial side down in the center of each tube, and pure PDA plugs were used as control. Prior to seedling inoculation, fungi were cultured in the medium for 3–7 days.

The 8-days-old rice seedlings were transplanted to the tubes that were pre-inoculated with fungi (five seedlings per tube). Three–five replications of each treatment were set up. Tubes were double sealed with parafilm and arranged randomly and finally kept in the growth chamber at 28 ± 1°C with a photoperiod of 12 h of light/12 h darkness. After incubation for 15 days, fresh biomass, plant height, root length were measured. Statistical analysis was performed using GraphPad Prism v6.0 (GraphPad, San Diego, CA, USA). Statistically significant differences between treatment and control groups were analyzed using multiple *t*-tests comparison.

### Microscopic Observation of Fungal Colonization Pattern in Roots

Trypan blue staining method was used to confirm whether the pleosporalean fungi colonize the inner roots endophytically (Padamsee et al., 2016). Briefly, the roots were cleaned and fixed in 50% ethanol for 24 h. Then the samples were rinsed three times with sterilized deionized water and then soaked into 5% potassium hydroxide (KOH) for 2–3 h in a water bath at 90°C. Subsequently, the roots were acidified by 2% lactic acid for 1–2 min followed by strained with 0.05% (w/v) trypan blue (a mixture of 1:1:1 lactic acid/glycerol/distilled water) for 10 h. Finally, the roots were placed into 50% glycerin for 24 h to de-stain. The hyphal structures in roots were viewed with light microscope (Zeiss, Axio Scope A1) using differential interference contrast (DIC) illumination and an AxioCam MRc5 camera and Zen software.

## Results

### Identification and Molecular Phylogenetic Appraisal of Plant-Associated Pleosporalean Fungi

As shown in **Table 1**, a preliminary BLAST analysis of ITS and LSU sequences from the 27 fungal strains isolated from halophyte roots, seeds, and rhizosphere showed that they comprised a diverse group of pleosporalean taxa.

**FIGURE 2:**
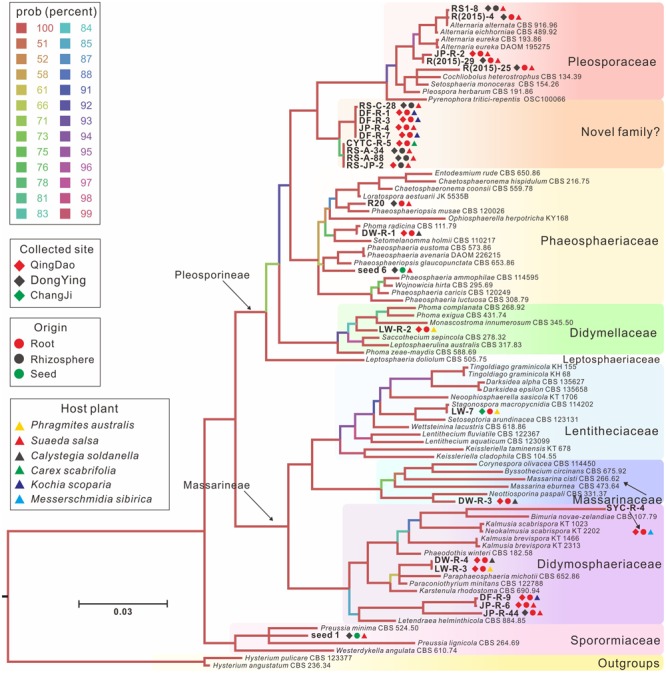
**A majority rule Bayesian phylogram showing the relationships between our isolates and currently described pleosporalean fungi, based on the concatenated alignment of orthologous genes encoding ribosomal RNAs from two subunits, the large subunit (LSU) and small subunit (SSU), and a large fragment of the transcription elongation factor 1-α gene (*tef1*)**. The color of branch corresponded to the posterior probabilities percentage. Accession numbers of the three genes used for the phylogram were given in **Table 1** and Supplementary Table S1. Different symbols showing collection sites, origin materials and host plants for each isolate were also indicated.

To identify species level taxonomy and determine the relationships among our isolates and known pleosporalean species, we constructed a phylogeny using a multi-locus DNA sequence dataset (SSU, LSU, and *tef1*) from 89 taxa. *Hysterium angustatum* and *H. pulicare* (Hysteriales, Dothideomycetes) served as outgroup taxa (**Figure 2**). The analysis indicates that the pleosporalean fungi obtained in our work belong to seven distinct families including Phaeosphaeriaceae, Pleosporaceae, Didymellaceae, Sporormiaceae, Didymosphaeriaceae, Massarinaceae, and Lentitheciaceae, and were distributed across two suborders (Pleosporineae and Massarineae). Most isolates belong to the families Phaeosphaeriaceae, Pleosporineae, and Didymosphaeriaceae. It should be especially noted that, nine isolates isolated from three host plants and two compartments (rhizosphere and endosphere) formed a strongly supported clade sister to Phaeosphaeriaceae and Pleosporaceae, suggesting their potentially taxonomic novelty. Furthermore, LSU and/or ITS sequences of some isolates (such as SYC-R-4, JP-R-6, DF-R-9, JP-R-44, and LW-R-2) did not match any known species, and formed several strongly supported separate clades. This suggests that they are potentially new genera and that likely many more taxa to be discovered in this order. The three individual gene trees also yielded very similar topologies (**Supplementary Figure S1**).

### Comparison of the Utilization of Inorganic and Organic Nitrogen in Pure Cultures

**FIGURE 3:**
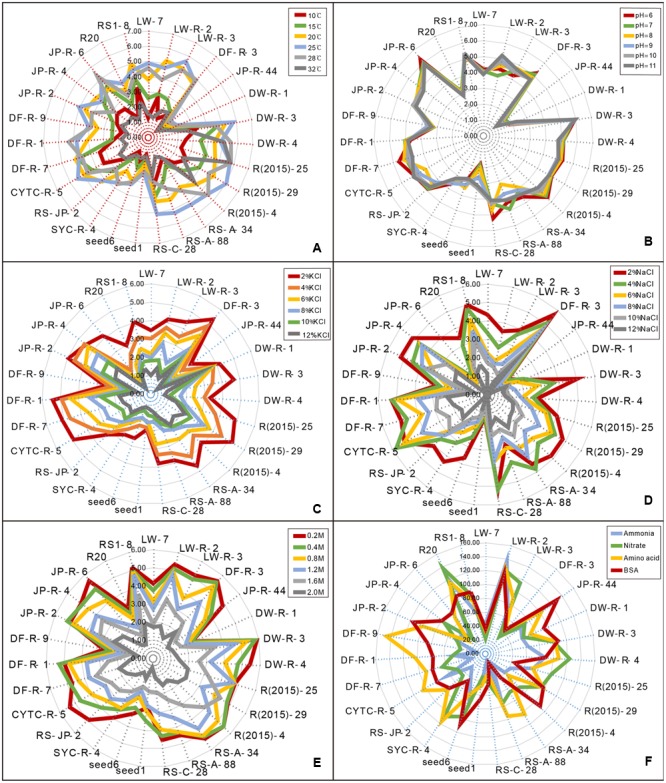
**Radar charts showing the different sensitivity of pleosporalean fungal growth to diverse stress conditions and their nitrogen utilization pattern.** Fungal growth was estimated by measured parameters of diameter on PDA plates (for stress assays) or dry weight of the biomass production in liquid medium (for nitrogen utilization assay). **(A)** Effects of different temperature on the growth of the isolates. **(B)** Effects of different pH level on the growth of the isolates. **(C,D)** Effects of a range of salt (NaCl and KCl) concentrations on the growth of the isolates. **(E)** Effects of the non-ionic osmotic stress (sorbitol) on the growth of the isolates. **(F)** Variable nitrogen utilization pattern. (NH_4_)_2_HPO_4_ and KNO_3_ were used as inorganic N, while a mixture of amino acids and Bovine Serum Albumin (BSA) as organic N.

All isolates investigated utilize both inorganic and organic N in liquid cultures. While final strain biomass was variable at different N sources (**Figure 3F**), a total of 21 isolates preferred organic N over inorganic N (**Supplementary Figure S2**, *P* < 0.05). Isolates of the suborder Pleosporineae produced the highest biomass on organic N. Strains LW-7 (Lentitheciaceae sp.) and LW-R-2 (*Epicoccum* sp.) accumulated the greatest biomass when NH_4_^+^ was the sole N-source, while the remaining four strains of R20 (*Phaeosphaeriopsis* sp.), R(2015)-25 (*Curvularia* sp.), LW-R-3 (*Paraconiothyrium* sp.), and DW-R-4 (*Paraconiothyrium* sp.) produced the greatest biomass on nitrate.

### Environmental Stress Response in the Plant-Associated Pleosporalean Fungi

#### Temperature

Effects of temperature on growth of the pleosporalean fungi were shown in **Figure 3A**. Although the growth rate varied among isolates, 25°C was the optimal temperature for most fungi. Of these, the isolates of JP-R-44, DW-R-1 and seed1 grew much more slowly than other isolates under the same conditions. An exception was the isolate R(2015)-25 (*Curvularia* sp.), which still grows well at 32°C.

#### pH

With the exception of DW-R-1 and JP-R-44, the majority of strains grow well over a wide range of pH level ranging from 6 to 11 (**Figure 3B**) suggesting that they are highly alkali-resistant. Isolates (LW-R-2 and CYTC-R-5) performed well at high pH suggesting adaptation to the alkali environments as is characteristic of alkalophilic fungi (Horikoshi, 1999).

#### Salt Stresses

All isolates were sensitive to salt. Overall, their growth rates were clearly inhibited with increasing concentrations of KCl or NaCl (**Figures 3C,D**), although the mycelium remained viable. Growth in the presence of increased concentrations of NaCl was considerably more inhibited than KCl at the same concentration. Only a small number of isolates were able to grow at higher NaCl concentrations. In contrast, several of them (including LW-R-2, JP-R-44, DW-R-1, and seed1) could still grow at the highest KCl concentration tested.

#### Non-ionic Osmotic Stress

Similar to salt sensitivity, increased sorbitol concentrations also inhibited fungal growth (**Figure 3E**), but the effect was much less pronounced in comparison to the ionic salt stress.

### Nitrogen Status Influences the Outcome of Plant–Fungal Interactions

**FIGURE 4:**
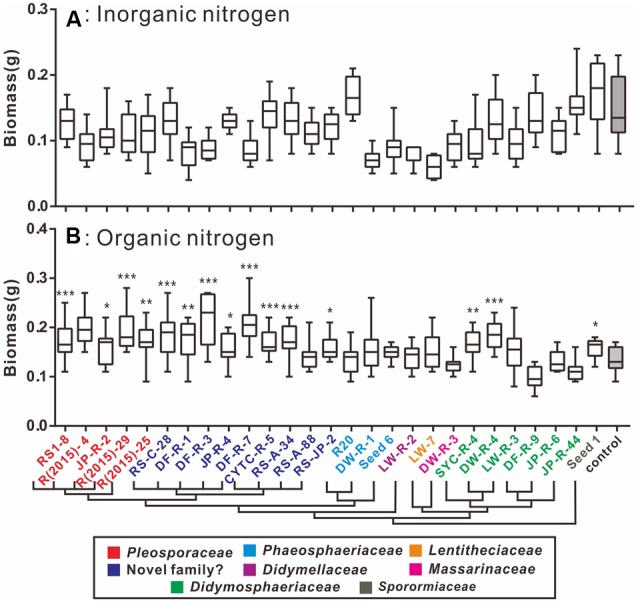
**Box-plots showing the plant biomass of fungal-treated and control groups supplied with inorganic (A)** and organic nitrogen **(B)**. **(A)** In the presence of inorganic N, the majority of isolates had non-significant positive influences on the biomass of the seedlings compared to the uninoculated control group. **(B)** In the presence of organic N, significant differences to the control in biomass were evaluated for the seedlings inoculated with the tested isolates, which were denoted by ^∗^*P* < 0.05, ^∗∗^*P* < 0.01, ^∗∗∗^*P* < 0.0001. Horizontal bars within the box indicated the median value of the data, and the outer vertical bars represented the maximum and minimum values of the data. A cluster tree modified from **Figure 2** was added to clarify the linkage between the phylogeny within the fungal isolates and their functions.

**FIGURE 5:**
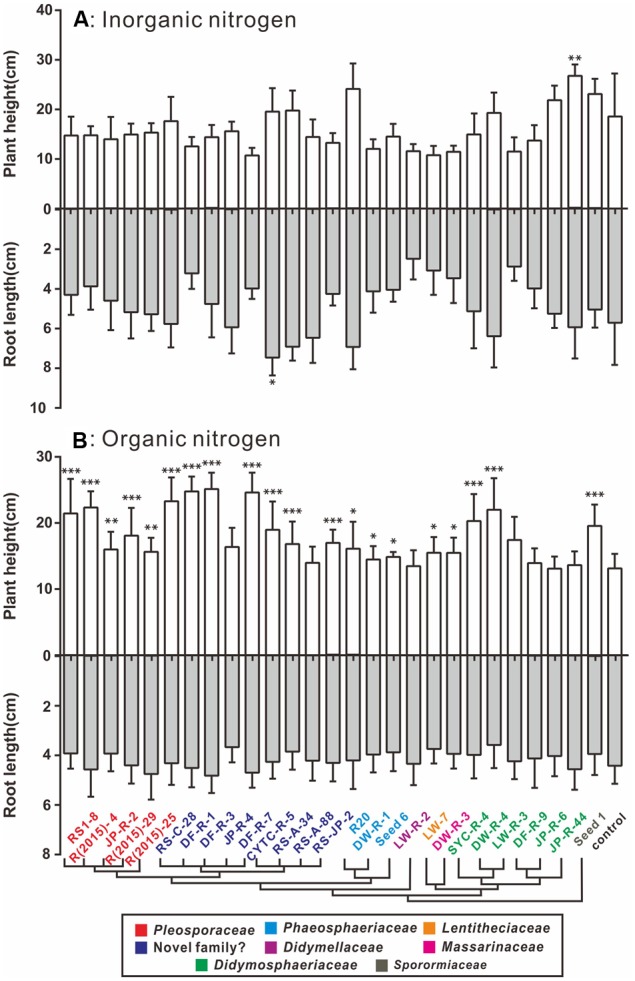
**Plant height and root length of fungal-treated groups and control groups supplied with inorganic (A)** and organic nitrogen **(B)**. **(A)** In the presence of inorganic N, most inoculated treatments showed inhibitory impact on plant height and root length. **(B)** In the presence of organic N, several isolates significantly promoted the growth of plant height (*P* < 0.0001) compared to the uninoculated control group, and did not significantly increase root growth. Values for inoculated plants differing from the control group were denoted by ^∗^*P* < 0.05, ^∗∗^*P* < 0.01, ^∗∗∗^*P* < 0.0001. Bars indicated standard error in the histogram.

A plant–fungi co-cultivation system was established to explore potential beneficial effects of pleosporalean fungi on plant growth. In view of the importance of the type of nitrogen in plant performance and productivity, we investigated whether the nitrogen source would influence the outcome of plant–fungal interactions (Upson et al., 2009; Newsham, 2011). Our preliminary data established that none of the isolates cause disease symptoms in rice seedlings (data not shown). As shown in **Figure 4**, plants co-cultured with fungi did not have any positive effects on plant biomass, and in some cases even negatively affected the growth when inorganic nitrogen sources (NH_4_NO_3_ and KNO_3_) were used. While the root length of rice seedlings treated with CYTC-R-5 and plant height of JP-R-44 treated seedlings were enhanced (multiple *t*-tests comparison, df = 26; *P* < 0.05) (**Figure 5**), yet no significant differences in plant biomass were observed (**Figure 4**).

When organic nitrogen was provided, almost all isolates increased plant biomass production (**Figure 4**). Isolates DF-R-3 (*Phaeosphaeriaceae* sp.), DF-R-7 (*Phaeosphaeriaceae* sp.), DW-R-4 (*Paraconiothyrium* sp.), R(2015)-29 (*Alternaria* sp.), and R(2015)-4 (*Alternaria* sp.) significantly promoted seedling growth (multiple *t*-tests comparison, df = 24; *P* < 0.0001). Also, 12 other groups clearly improved plant development (multiple *t*-tests comparison, df = 24; *P* < 0.05). The data also showed that fungal induced plant biomass increases are mainly due to the accumulation of above-ground biomass as indicated by increased plant height (multiple *t-*tests comparison, df = 24; *P* < 0.05) and not root biomass (**Figure 5**).

### Fungal Colonization Pattern in Plant Roots

**FIGURE 6:**
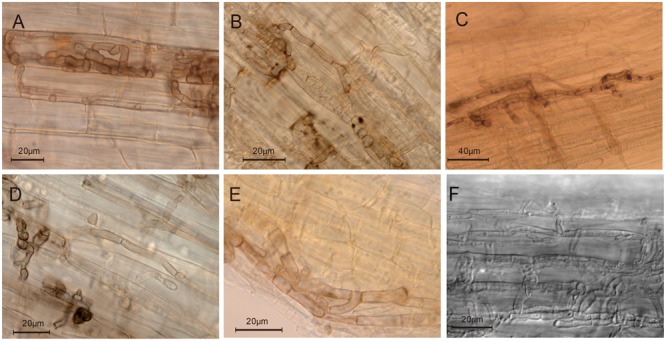
**Endophytic colonization of plant roots by six pleosporalean taxa.** Intracellular hyaline and septate hyphae were observed in the root tissues which were inoculated with these endophytes [RS-A-34, RS-C-28, RS-A-88, R(2015)-25, DW-R-4 and R20] **(A–F)** with the light microscopy.

The endophytic colonization of rice roots by the pleosporalean fungi was further investigated microscopically. Six isolates [RS-A-34, RS-C-28, RS-A-88, R(2015)-25, DW-R-4 and R20] (**Figures 6A–F**) successfully colonized the inner root tissue as shown by the distribution of an abundant number of hyphae in the cortex and epidermis cells, both inter- and intracellularly. Melanized microsclerotia-like structures were formed upon inoculation with the isolate RS-A-34 (**Figure 6A**), showing the typical characters of DSEs. Root cells were filled with microsclerotia-like structures. Although inoculation of the isolates RS-C-28, RS-A-88, R(2015)-25 did not form the typical microsclerotia-like structures in roots, initiation and development of chlamydospore-like structures have likely occurred. The remaining isolates colonized rice roots only on the surface, because no fungal structures were found inside root cells. Roots inoculated with the isolates DW-R-1 and R(2015)-29 became brown to dark, but no hyphae were seen in roots.

## Discussion

Apart from AMF and EMF, NMF have now been recognized as an important component of the root-associated mycobiome (Khidir et al., 2010; Andrade-Linares and Franken, 2013; Zuccaro et al., 2014). While there is accumulating evidence regarding the diversity and structure of NMF, their basic physiology and extended effects on plants are not well characterized, especially for plant-associated NMF under extreme conditions. In this work, we characterized halophyte-associated fungi from three geographic areas, six halophyte plant species and three habitats (rhizosphere, root, and seed endosphere), and found that the pleosporalean taxa can be frequently captured. This implies that they are generalist endophytes and/or epiphytes in high salinity environments, and might also mean that this group of fungi is the easiest to isolate and culture under the experimental conditions. This is consistent with other studies where pleosporalean fungi were found to be the dominant colonizers in halophytes (El-Morsy, 2000; Sun et al., 2011; Okane and Nakagiri, 2015) and plants grown in arid conditions (Porras-Alfaro et al., 2008; Khidir et al., 2010). Especially the genera *Pleospora, Alternaria*, and *Phoma* were often recorded. Here we show that all pleosporalean strains isolated from our study can be categorized into seven families. Some of them are newly discovered taxa as several clades separated from known fungal taxa by long and well-supported branches (**Figure 2**). To the best of our knowledge, there is only a single report on a systematic morphological and phylogenetic analysis of diverse pleosporalean DSEs available (Knapp et al., 2015). Consequently, the ubiquity, diversity and novelty of plant-associated pleosporalean fungi in adverse environments will not only provide good phylogenetic resolution within the Pleosporales, but also provide the impetus to elucidate their basic physiology and roles in plant fitness.

To our knowledge, this study presents the first *in vitro* experimental evidence of the ability of pleosporalean fungi to adapt to a series of environmental stresses. Our data showed that the tested isolates are sensitive to ionic (imposed by NaCl and KCl) and non-ionic osmolytes (imposed by sorbitol) with varying degrees, which is consistent with previous observations (Larsen, 1986; Nikolaou et al., 2009; Samapundo et al., 2010). Most of our isolates were more negatively affected by NaCl stress than KCl or sorbitol stress. It is possible that Na^+^ is poorly taken up by fungi, and could in fact cause alkaline stress, while K^+^ can be easily absorbed and would not accumulate as KOH in the medium (Larsen, 1986). More importantly, some fungi may also utilize K^+^ for surviving in unfavorable conditions (Larsen, 1986). Despite their salt sensitivity, few of them still can grow and survive in 12% NaCl (approximately 2 M NaCl). This is consistent with earlier data that the pleosporalean fungi isolated from halophytes are more likely halotolerant but not halophilic (Rodriguez et al., 2008; Lucero et al., 2011; Maciá-Vicente et al., 2012). Maciá-Vicente et al. (2012) further speculated that rhizospheric soil fungi may be more tolerant to salt stress than endophytes, as endophytes are protected within plant roots from harsh soil conditions. Our data did not, however, support this hypothesis since there was no significant difference of growth pattern under salinity stress between endophytes and rhizospheric fungi in our conditions. All fungi tested could grow well at high pH, which may imply that both soil and host provide a well-conditioned environment with high alkali for the rhizospheric and endophytic pleosporalean fungi. Taken together, the evidence from the present study suggests that the ability of coping with multiple ecological stresses in pleosporalean fungi should be taken into consideration for their utilization in saline-alkaline soils.

It has been known that different nitrogen sources can affect ectomycorrhizal and DSEs fungal growth and biomass accumulation (Yamanaka, 1999; Rosling et al., 2004; Mandyam et al., 2010). Our results demonstrated that fungal biomass formation on different inorganic and organic substrates significantly varied among species, but most preferred amino acids over inorganic nitrogen sources presumably due to energy and carbon savings for amino acid biosynthesis. Mandyam et al. (2010) also found that several DSEs produced more biomass in the organic N (Gly) than in the inorganic N. Besides that, fungi also can use them as carbon sources for growth. The effective utilization of BSA in many isolates may reflect the occurrence of fungal-derived proteolytic enzymes for hydrolyzing proteins (Leake and Read, 1990; Bizabani and Dames, 2016). We suggest that this trait maybe related to their host plants N nutrition (Cairney and Meharg, 2003) (see further discussion below).

The influence of NMFs on growth and stress tolerance of plants are now beginning to be revealed. It has often been hypothesized that DSEs confer plant drought tolerance and nutrient acquisition (Porras-Alfaro et al., 2008), whereas other NMFs have been reported to have weak or even negative effects on plant growth (Jumpponen, 2001; Kageyama et al., 2008; Dovana et al., 2015). It is well-known that a number of factors determine the outcome of plant–fungal interactions, including plant genotype, the genotype and virulence of the fungi as well as the environmental conditions and nutrient status of the soil (Schulz and Boyle, 2005; Singh et al., 2011; Murphy et al., 2014). The co-cultivation assay confirmed that our isolates colonized rice seedlings asymptomatically. This suggests that the inhibition of plant growth caused by pleosporalean fungi probably results from uncontrolled fungal growth, but not from the fungal virulence factors (mainly mycotoxins) (Vahabi et al., 2013).

Since plants obtain their carbon from carbon dioxide, N is the major nutrient they have to retrieve from the soil (Blair et al., 1998). For most plants, inorganic nitrogen compounds are the major source of soluble N (Roberts et al., 2009). In line with previous studies, our data strongly indicates that the nitrogen source influences plant–fungal interactions (Newsham, 2011). *In vitro* closed co-cultivation system under axenic conditions has been often used for studying plant-NMF interactions. In this case, the most frequently used substrates supporting plant and fungal growth are the MS medium (half strength or one-tenth strength) (Kageyama et al., 2008; Junker et al., 2012) and one-tenth strength of Marx-Melin-Norkrans (MMN) medium (Keim et al., 2014), which contains N in the inorganic form. Using these media, researchers found that the majority of fungal endophytes adversely affected plant growth and health, and a few of them were even pathogenic to plants (Jumpponen, 2001; Dovana et al., 2015). However, plant growth promotion can be measured in this experimental system if the fungi prove to produce auxin-like compounds *in vitro* (Sirrenberg et al., 2007; Contreras-Cornejo et al., 2009; Redman et al., 2011) despite clear evidence of *in situ* hormone production *in planta* by endophytes is yet to be elucidated. In the presence of organic N condition, most pleosporalean fungi strongly enhance plant biomass accumulation compared to the presence of inorganic N. This raises the question of why and how the N source influences the interaction with plant roots. It is known that tryptophan (Trp) is a precursor for auxin biosynthesis in fungi and plants (Redman et al., 2011). However, Trp is not present in the range of organic N sources we tested in the context of this study. Those which are present (Gly, Val, Glu, Phe, and Leu) are not known as precursors for the synthesis of plant-growth-promoters. Rather, we propose the possibility that the amino acids will become mineralized upon fungal colonization and therefore available for plant uptake. This hypothesis is supported by earlier reports showing that a wide range of DSE provide more benefits to plant in the presence of organic N than in the presence of inorganic N (Mandyam and Jumpponen, 2005; Upson et al., 2009; Alberton et al., 2010; Newsham, 2011; Mahmoud and Narisawa, 2013). DSE has been shown to synthesize proteolytic enzymes, which can mineralize the organic N compounds into the free inorganic N (Caldwell et al., 2000; Bizabani and Dames, 2016). This work further extends our view that apart from DSE, the rhizospheric and seed endophytic fungi may also possess a similar functional trait.

It has been reported that insect pathogenic fungi and EMF can transport insect-derived organic nitrogen into the plant roots (Klironomos and Hart, 2001; Behie et al., 2012, 2013). More broadly, some rhizobacterial symbionts of plants also secrete proteases and degrade denatured proteins and scavenge organic nitrogen from soil (White et al., 2015). We might say to strengthen this section that bacterial/fungal chitinase and protease activities are known to participate to the N cycle and are crucial for decomposition of soil organic nitrogen (Heinonsalo et al., 2015; Rineau et al., 2015; Knapp and Kovács, 2016). Hence, it appears likely that diverse plant-associated fungi and bacteria are important players in the soil nitrogen cycle (Behie et al., 2013). As the excessive use of inorganic nitrogen poses a great threat to natural ecosystems and crop yield (Tilman et al., 2002), our findings underscore the enormous potential for utilizing organic N mineralizing microbes in sustainable agriculture. Indeed, some of our isolates have also been shown to promote the growth of trees (e.g., American sweetgum (*Liquidambar styraciflua*) seedlings) under organic N condition (data not shown), thus suggesting the application of this system over a wider range of plants.

## Conclusion

Our work provides new insights into the biodiversity of the widespread pleosporalean fungi associated with halophytes. Future direction will thus be focused on addressing whether these fungi are involved in plant salt tolerance. Symbiotic interactions between plant and pleosporalean fungi may serves as a new model for studying fungal-mediated plant growth and stress tolerance.

## Author Contributions

ZY conceived and designed the experiment. YQ performed the experiment. ZY and YQ wrote the paper. XP helped to isolate the pleosporalean fungi. ID and KC constructed the phylogenetic tree. CK and JL revised the paper.

## Conflict of Interest Statement

The authors declare that the research was conducted in the absence of any commercial or financial relationships that could be construed as a potential conflict of interest.

## Footnotes

1 https://blast.ncbi.nlm.nih.gov/Blast.cgi

2 http://tree.bio.ed.ac.uk/software/figtree/

## Supplementary Material

FIGURE S1 **Bayesian consensus phylograms for individual genetic regions (LSU, SSU, and *tef1*) showing the relationships between our isolates and currently described pleosporalean fungi.** The color of branch corresponded to the posterior probabilities percentage.

FIGURE S2 **Fungal biomass production (mean ± standard error) of 27 tested isolates under four different N sources including ammonium (NH_4_^+^), nitrate (NO_3_^-^), a mixture of five amino acids and BSA.** Different letters above columns indicated the significant differences (multiple *t*-tests comparison, *P* < 0.05).
