# Supplementary figures and images for: Diverse Plant-Associated Pleosporalean Fungi from Saline Areas: Ecological Tolerance and Nitrogen-Status Dependent Effects on Plant Growth
Source: Front Microbiol. 2017 Feb 6;8:158. doi: 10.3389/fmicb.2017.00158 (PMC5292420; doi:10.3389/fmicb.2017.00158)

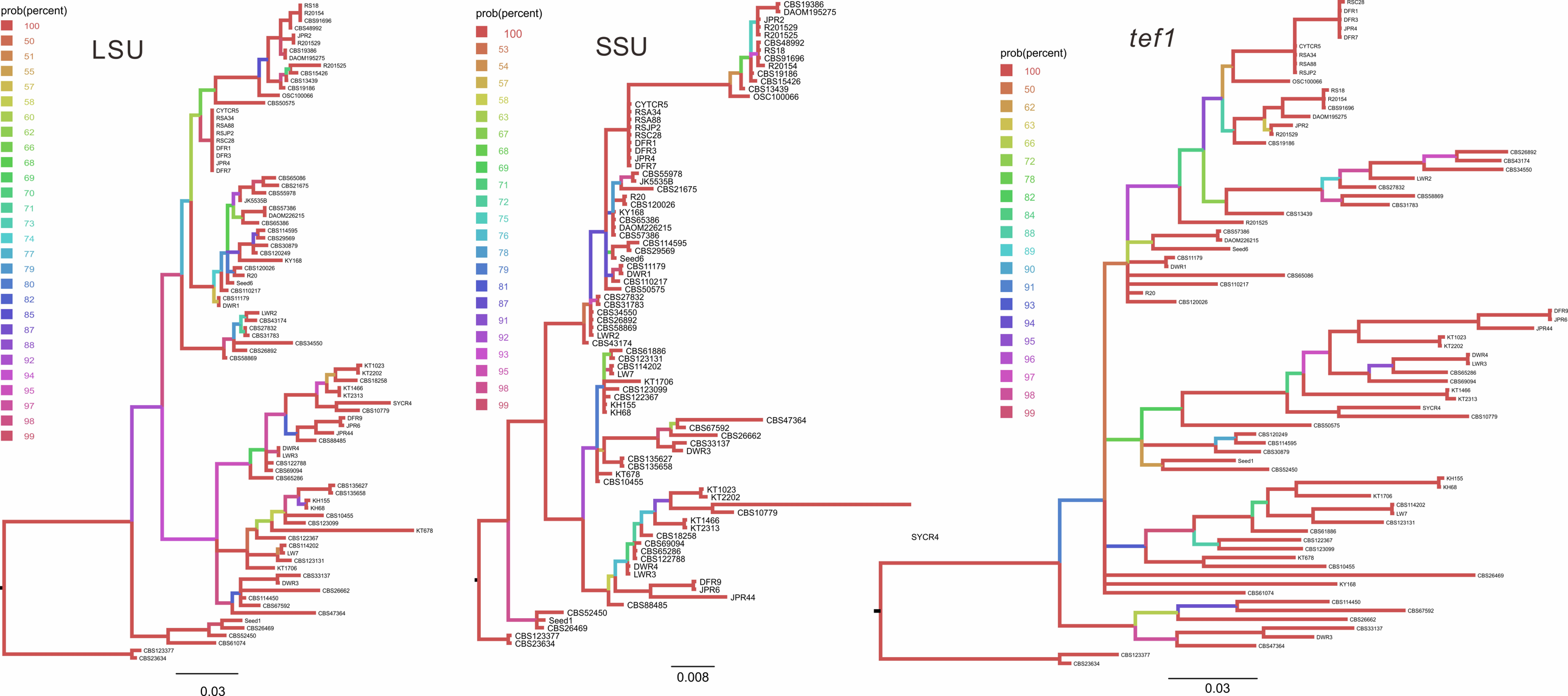

Supplement: FIGURE S1 — Bayesian consensus phylograms for individual genetic regions (LSU, SSU, and tef1) showing the relationships between our isolates and currently described pleosporalean fungi. The color of branch corresponded to the posterior probabilities percentage. [file Image_1.JPEG]

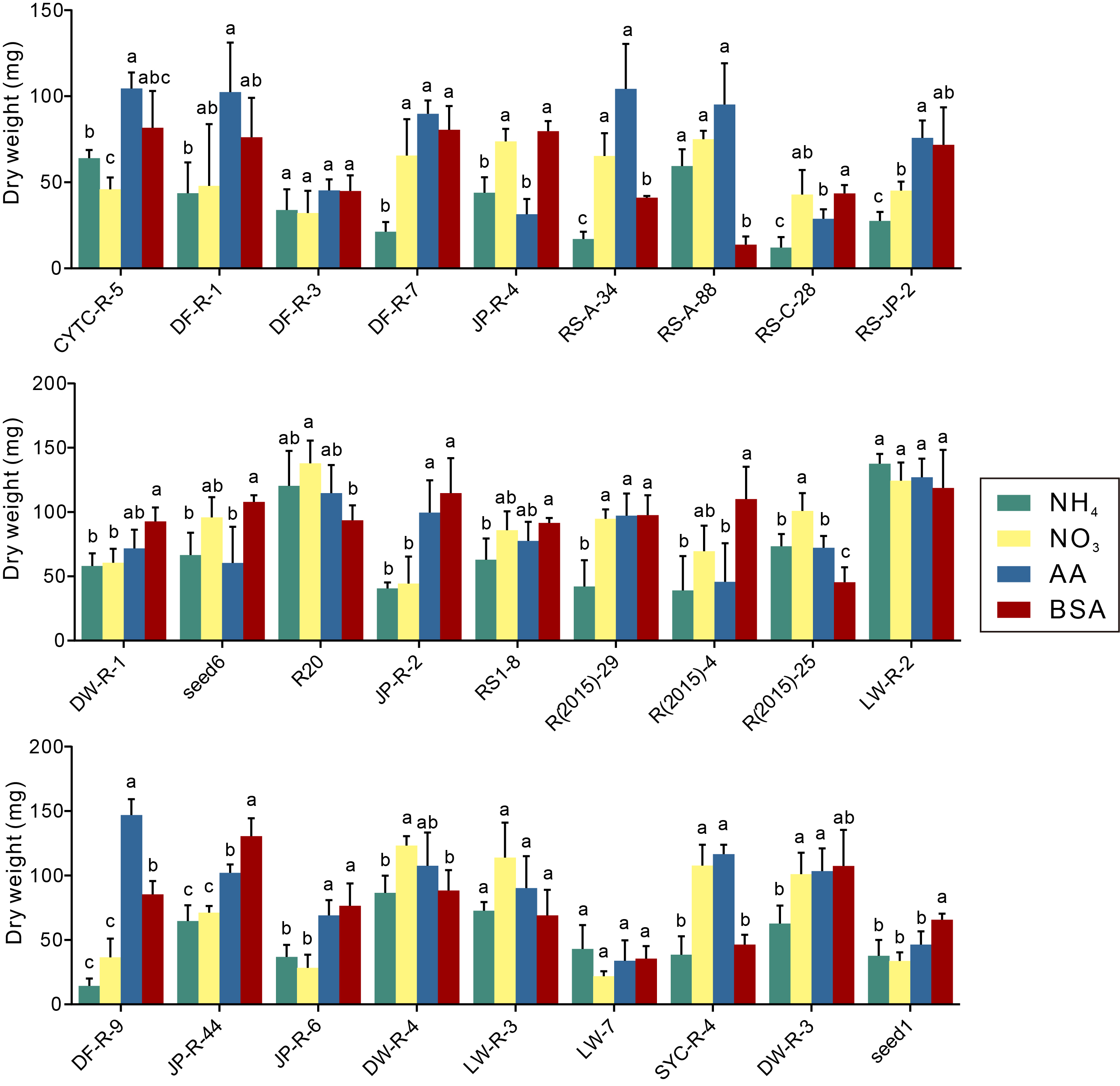

Supplement: FIGURE S2 — Fungal biomass production (mean ± standard error) of 27 tested isolates under four different N sources including ammonium (NH4+), nitrate (NO3-), a mixture of five amino acids and BSA. Different letters above columns indicated the significant differences (multiple t-tests comparison, P < 0.05). [file Image_2.JPEG]
