# Supplementary material for: Diverse Plant-Associated Pleosporalean Fungi from Saline Areas: Ecological Tolerance and Nitrogen-Status Dependent Effects on Plant Growth
Source: Front Microbiol. 2017 Feb 6;8:158. doi: 10.3389/fmicb.2017.00158 (PMC5292420; doi:10.3389/fmicb.2017.00158)
Supplement: Supplementary file 3 [file Table_1.DOCX]

**Table S1** Culture collection and GenBank accession numbers of three loci used for phylogeny construction.

| **Species** | **Isolate code** | **Host** | **GenBank accession no.** | | | **References** |
| --- | --- | --- | --- | --- | --- | --- |
|  |  |  | **LSU** | **SSU** | ***tef1*** |  |
| *Alternaria alternata* | CBS 916.96 | *Arachis hypogaea* | DQ678082 | KC584507 | DQ677927 | Woudenberg et al. (2013) |
| *Alternaria eichhorniae* | CBS 489.92 | *Eichhornia crassipes* | KP124579 | KP125049 | --- | Woudenberg et al. (2015) |
| *Alternaria eureka* | CBS 193.86 | *Medicago rugosa* | KC584331 | KC584589 | --- | Woudenberg et al. (2013) |
| *Alternaria eureka* | DAOM 195275 | *---* | DQ678044 | DQ677994 | DQ677883 | Schoch et al. (2006) |
| *Bimuria novae-zelandiae* | CBS 107.79 | *---* | AY016356 | AY016338 | DQ471087 | Lumbsch et al. (2001) |
| *Byssothecium circinans* | CBS 675.92 | *---* | GU205217 | GU205235 | GU349061 | --- |
| *Chaetosphaeronema coonsii* | CBS 559.78 | *Malus sylvestris* | EU754196 | EU754097 | --- | de Gruyter et al. (2009) |
| *Chaetosphaeronema hispidulum* | CBS 216.75 | *Anthyllis vulneraria* | KF251652 | EU754045 | --- | Quaedvlieg et al. (2013) |
| *Cochliobolus heterostrophus* | CBS 134.39 | *---* | AY544645 | AY544727 | DQ497603 | *---* |
| *Corynespora olivacea* | CBS 114450 | *---* | GU301809 | --- | GU349014 | Schoch et al. (2009) |
| *Darksidea alpha* | CBS 135627 | *Bromus tectorum* | KP184005 | KP184044 | --- | Knapp et al. (2015) |
| *Darksidea epsilon* | CBS 135658 | *Stipa borysthenica* | KP184029 | KP184070 | --- | Knapp et al. (2015) |
| *Entodesmium rude* | CBS 650.86 | *---* | GU301812 | --- | GU349012 | Schoch et al. (2009) |
| *Kalmusia brevispora* | KT 1466 | *Sasa sp., culms* | AB524600 | AB524459 | AB539112 | Tanaka et al. (2009) |
| *Kalmusia brevispora* | KT 2313 | *Sasa kurilensis, culms* | AB524601 | AB524460 | AB539113 | Tanaka et al. (2009) |
| *Kalmusia scabrispora* | KT 1023 | *Phyllostachys bambusoides, culms* | AB524593 | AB524452 | AB539106 | Tanaka et al. (2009) |
| *Karstenula rhodostoma* | CBS 690.94 | *---* | GU301821 | GU296154 | GU349067 | Schoch et al. (2009) |
| *Keissleriella cladophila* | CBS 104.55 | *---* | GU301822 | GU296155 | GU349043 | Schoch et al. (2009) |
| *Keissleriella taminensis* | KT 678 | herbaceous plant | AB807597 | AB797307 | AB808575 | Tanaka et al. (2015) |
| *Lentithecium aquaticum* | CBS 123099 | *---* | GU301823 | GU296156 | GU349068 | Schoch et al. (2009) |
| *Lentithecium fluviatile* | CBS 122367 | *---* | GU301825 | GU296158 | GU349074 | Schoch et al. (2009) |
| *Leptosphaeria doliolum* | CBS 505.75 | *Urtica dioica* | GQ387576 | GQ387515 | GU349069 | de Gruyter et al. (2010) |
| *Leptosphaerulina australis* | CBS 317.83 | *Eugenia aromatica* | EU754166 | EU754067 | GU349070 | de Gruyter et al. (2009) |
| *Letendraea helminthicola* | CBS 884.85 | *---* | AY016362 | AY016345 | --- | Lumbsch et al. (2001) |

**Table S1** (Continued)

| **Species** | **Isolate code** | **Host** | **GenBank accession no.** | | | **References** |
| --- | --- | --- | --- | --- | --- | --- |
|  |  |  | **LSU** | **SSU** | ***tef1*** |  |
| *Loratospora aestuarii* | JK 5535B | *---* | GU301838 | GU296168 | --- | Schoch et al. (2009) |
| *Massarina cisti* | CBS 266.62 | *---* | AB807539 | AB797249 | AB808514 | Tanaka et al. (2015) |
| *Massarina eburnea* | CBS 473.64 | *---* | GU301840 | GU296170 | GU349040 | Schoch et al. (2009) |
| *Monascostroma innumerosum* | CBS 345.50 | *---* | GU301850 | GU296179 | GU349033 | Schoch et al. (2009) |
| *Neokalmusia scabrispora* | KT 2202 | *Phyllostachys bambusoides, culms* | AB524594 | AB524453 | AB539107 | Tanaka et al. (2009) |
| *Neoophiosphaerella sasicola* | KT 1706 | *Sasa kurilensis, culms* | AB524599 | AB524458 | AB539111 | Tanaka et al. (2009) |
| *Neottiosporina paspali* | CBS 331.37 | *Paspalum notatum* | EU754172 | EU754073 | GU349079 | de Gruyter et al. (2009) |
| *Ophiosphaerella herpotricha* | KY168 | Bermudagrass | KP690987 | KP690987 | KP691017 | *---* |
| *Paraconiothyrium minitans* | CBS 122788 | *---* | EU754173 | EU754074 | --- | de Gruyter et al. (2009) |
| *Paraphaeosphaeria michotii* | CBS 652.86 | *---* | JX496216 | GQ387520 | GU456266 | Verkley et al. (2014) |
| *Phaeodothis winteri* | CBS 182.58 | *---* | GU301857 | GU296183 | --- | Schoch et al. (2009) |
| *Phaeosphaeria ammophilae* | CBS 114595 | *---* | GU301859 | GU296185 | GU349035 | Schoch et al. (2009) |
| *Phaeosphaeria avenaria* | DAOM 226215 | *---* | AY544684 | AY544725 | DQ677885 | Lutzoni et al. (2004) |
| *Phaeosphaeria caricis* | CBS 120249 | *---* | GU301860 | --- | GU349005 | Schoch et al. (2009) |
| *Phaeosphaeria eustoma* | CBS 573.86 | *---* | DQ678063 | DQ678011 | DQ677906 | Schoch et al. (2006) |
| *Phaeosphaeria luctuosa* | CBS 308.79 | *---* | GU301861 | --- | GU349004 | Schoch et al. (2009) |
| *Phaeosphaeriopsis glaucopunctata* | CBS 653.86 | *Ruscus aculeatus* | KF251702 | GQ387531 | --- | Quaedvlieg et al. (2013) |
| *Phaeosphaeriopsis musae* | CBS 120026 | *---* | GU301862 | GU296186 | GU349037 | Schoch et al. (2009) |
| *Phoma complanata* | CBS 268.92 | *Angelica sylvestris* | EU754180 | EU754081 | GU349078 | de Gruyter et al. (2009) |
| *Phoma exigua* | CBS 431.74 | *Solanum tuberosum* | EU754183 | EU754084 | GU349080 | de Gruyter et al. (2009) |
| *Phoma radicina* | CBS 111.79 | *Malus sylvestris* | KF251676 | EU754092 | GU349076 | Quaedvlieg et al. (2013) |
| *Phoma zeae-maydis* | CBS 588.69 | *Zea mays* | EU754192 | EU754093 | GU349082 | de Gruyter et al. (2009) |
| *Pleospora herbarum* | CBS 191.86 | *---* | DQ247804 | DQ247812 | DQ471090 | Schoch et al. (2006) |

**Table S1** (Continued)

| **Species** | **Isolate code** | **Host** | **GenBank accession no.** | | | **References** |
| --- | --- | --- | --- | --- | --- | --- |
|  |  |  | **LSU** | **SSU** | ***tef1*** |  |
| *Preussia lignicola* | CBS 264.69 | *---* | GU301872 | GU296197 | GU349027 | Schoch et al. (2009) |
| *Preussia minima* | CBS 524.50 | *---* | DQ678056 | DQ678003 | DQ677897 | Schoch et al. (2006) |
| *Pyrenophora tritici-repentis* | OSC100066 | *---* | AY544672 | AY544716 | DQ677882 | Lutzoni et al. (2004) |
| *Saccothecium sepincola* | CBS 278.32 | *---* | GU301870 | GU296195 | GU349029 | Schoch et al. (2009) |
| *Setomelanomma holmii* | CBS 110217 | *Picea pungens* | GQ387633 | GQ387572 | GU349028 | de Gruyter et al. (2010) |
| *Setoseptoria arundinacea* | CBS 123131 | *---* | GU456320 | GU456298 | GU456281 | Zhang et al. (2009) |
| *Setosphaeria monoceras* | CBS 154.26 | *---* | AY016368 | AY016352 | --- | Lumbsch et al. (2001) |
| *Stagonospora macropycnidia* | CBS 114202 | *---* | GU301873 | GU296198 | GU349026 | Schoch et al. (2009) |
| *Tingoldiago graminicola* | KH 155 | *Phragmites australis* | AB521745 | AB521728 | AB808562 | Hirayama et al. (2010) |
| *Tingoldiago graminicola* | KH 68 | *Phragmites australis* | AB521743 | AB521726 | AB808561 | Hirayama et al. (2010) |
| *Westerdykella angulata* | CBS 610.74 | *---* | DQ384105 | --- | GU371821 | Schoch et al. (2009) |
| *Wettsteinina lacustris* | CBS 618.86 | *---* | --- | DQ678023 | DQ677919 | Schoch et al. (2006) |
| *Wojnowicia hirta* | CBS 295.69 | *Lolium multiflorum* | EU754223 | EU754124 | --- | de Gruyter et al. (2009) |
